# PE-induced apoptosis in SMMC-7721 cells: Involvement of Erk and Stat signalling pathways

**DOI:** 10.3892/ijmm.2014.1777

**Published:** 2014-05-09

**Authors:** LI XUE, MING LI, TENG CHEN, HAIFENG SUN, JIE ZHU, XIA LI, FENG WU, BIAO WANG, JUPING LI, YANJIONG CHEN

**Affiliations:** 1 Forensic Medicine College of Xi’an Jiaotong University, Key Laboratory of the Health Ministry for Forensic Medicine, Key Laboratory of the Ministry of Education for Environment and Genes Related to Diseases, Xi’an, Shaanxi 710061, P.R. China; 2 Department of Cardiovascular Surgery, The First Affiliated Hospital of Medical College of Xi’an Jiaotong University, Xi’an, Shaanxi 710061, P.R. China; 3 Department of Immunology and Pathogenic Biology, Xi’an Jiaotong University School of Medicine, Xi’an, Shaanxi 710061, P.R. China; 4 Tumour Hospital of Shaanxi Province, Xi’an, Shaanxi 710061, P.R. China; 5 VIP Internal Medicine Department, Lanzhou University Second Hospital, Lanzhou, Gansu 730030, P.R. China; 6 Graduate Teaching and Experimental Centre, Xi’an Jiaotong University School of Medicine, Xi’an, Shaanxi 710061, P.R. China; 7 School of Public Security, Northwest University of Politics and Law, Xi’an, Shaanxi 710063, P.R. China

**Keywords:** phosphatidylethanolamine, apoptosis, SMMC-7721 cells, Erk, Stat

## Abstract

Emerging evidence indicates that the redistribution of phosphatidylethanolamine (PE) across the bilayer of the plasma membrane is an important molecular marker for apoptosis. However, the effect of PE on apoptosis and the underlying mechanism of PE remain unclear. In the current study, MTT and flow cytometric assays were used to examine the effects of PE on apoptosis in SMMC-7721 cells. The level of mitochondrial membrane potential (ΔΨm) and the expression of Bax, Bcl-2, caspase-3, phospho-Erk and phospho-Stat1/2 in SMMC-7721 cells that were exposed to PE were also investigated. The results showed that PE inhibited proliferation, caused G0/G1 phase cell cycle arrest and induced apoptosis in SMMC-7721 cells in a dose-dependent manner. Rhodamine 123 staining showed that the treatment of SMMC-7721 cells with different concentrations of PE for 24 h significantly decreased the level of ΔΨm and exerted dose-dependent effects. Using immunofluorescence and western blotting, we found that the expression of Bax was upregulated, whereas that of Bcl-2 was downregulated in PE-induced apoptotic cells. In addition, these events were accompanied by an increase in caspase-3 expression in a dose-dependent manner following PE treatment. PE-induced apoptosis was accompanied by a decrease in Erk phosphorylation and by the activation of Stat1/2 phosphorylation in SMMC-7721 cells. In conclusion, the results suggested that PE-induced apoptosis is involved in upregulating the Bax/Bcl-2 protein ratio and decreasing the ΔΨm. Moreover, the results showed that the Erk and Stat1/2 signalling pathways may be involved in the process of PE-induced apoptosis.

## Introduction

Phosphatidylethanolamine (PE) is an important phospholipid in mammalian membranes. PE is present primarily in the inner leaflet of the membrane bilayer in a viable, typical mammalian cell (1). PE is sustained in the bilayer configuration by interacting with other phospholipids in biological membranes. However, the reorganisation of membrane phospholipids could lead to the expression of the non-bilayer nature of PE and the induction of bilayer instability (2). Accumulating evidence has demonstrated that the translocation of PE to the cell surface in many distinct biological events (1). For instance, PE is exposed to the cell surface, thus providing a hallmark for detection in apoptotic cells (1). As a viable alternative, PE-binding probes have been shown to be selective in detecting dead and dying cells (3). Moreover, findings of a previous study indicated that exogenous PE induces apoptosis in hepatoma HepG2 cells (4). However, the effects of PE on apoptosis in SMMC-7721 cells and the underlying mechanism of PE remain unclear.

Mitochondria play a critical role in the regulation of apoptosis. Changes in the mitochondrial membrane potential (ΔΨm) may switch the committed cells to apoptotic death (5). Findings of previous studies demonstrated that mitochondrial dysfunction, which occurs during apoptosis, causes the release of cytochrome *c* and, thus, contributes to apoptosis (6,7). The Bcl-2 family proteins regulate the release of cytochrome *c* and other proteins through the outer mitochondrial membrane (OMM) (8,9). Some members of the Bcl-2 family inhibit apoptosis, such as Bcl-2, Bcl-xl, and Mcl-1, whereas Bax and Bak, activate apoptosis. Bax and Bak induced the release of cytochrome *c*, whereas anti-apoptotic Bcl-2 family members inhibited the release of cytochrome *c* (7). During the apoptotic process, the cytochrome *c* that is released from the mitochondria sequentially triggers a caspase cascade, which is characteristic of the apoptotic pathway, in which caspase-3 plays a dominant role (10). Therefore, a balance between pro-apoptotic (Bax/bad) and anti-apoptotic (Bcl-2/Bcl-xl) members of the Bcl-2 family proteins and their up- and downregulation usually determine whether cells undergo apoptosis or survive (11). However, the effect of the mitochondrial pathway on the PE-induced apoptosis of SMMC-7721 cells remains unclear.

As a member of the mitogen-activated protein kinases (MAPK) family, Erk is crucial in regulating cell growth and differentiation (12) and has been shown to act as an important modulator of various apoptosis-inducing signals in different systems (13,14). It was reported that the MEK/ERK signalling pathway regulated the expression of Bcl-2 (15). The aim of the present study was to investigate whether the Erk pathway is also involved in exogenous PE-induced apoptosis. Stat1 is partially phosphorylated by the Erk pathway (16), while the phosphorylation of Stat1 is generally associated with cell cycle arrest and apoptosis (17,18). Stat1 is important in the interferon-response, following various stressful stimuli that induce apoptotic or cell cycle checkpoint responses (16,19–22). Results of a previous study showed that the high expression of Stat1 and its activator (IFNγ) reduced the basal expression of the Bcl-2 promoter (23). To study the underlying mechanisms of PE-induced apoptosis, we investigated whether Erk and Stat1/2 signalling pathways were involved in PE-induced apoptosis in the hepatic cancer line SMMC-7721.

## Materials and methods

### Chemicals and reagents

Chemicals and cell culture reagents (RPMI-1640 medium, penicillin/streptomycin, and FBS) were obtained from Sigma (St. Louis, MO, USA) and Gibco Laboratories (Grand Island, NY, USA), respectively. An Annexin V-FITC Apoptosis Detection kit was obtained from BD Bioscience (Franklin Lakes, NJ, USA).

Antibodies to phosphospecific Erk1/2, Stat1, Stat2 and antibodies against Bax, Bcl-2, caspase-3 were purchased from Cell Signalling Technology, Inc. (Beverly, MA, USA). All the reagents were of analytical grade.

### Cell culture and treatment

SMMC7721 cells were provided by the Molecular Biology Centre of the First Affiliated Hospital, Xi’an Jiaotong University, China. SMMC-7721 cells were grown in RPMI-1640 medium, which was supplemented with 10% bovine serum albumin (BSA) and 1% penicillin/streptomycin in a humidified atmosphere of 95% air/5% CO_2_ at 37°C. The cells were cultured at different densities depending on the assay. The treatment was initiated with PE 24 h after plating. After the cells were treated with 0.125––1.0 mM/l PE for 6–48 h treatment, the cultures were terminated, and then adherent cells were collected for evaluation. The morphological change after exposure to PE was observed using a phase-contrast inverse microscope (DMIRBHC; Leica, Mannheim, Germany).

### Cell viability assay

Cells were cultured at a density of 2×10^4^ cells/well in a 96-well plate in RPMI-1640 medium. Cells were allowed to adhere and then treated with the indicated concentration of PE for 24 and 48 h. Subsequently, 20 μl of MTT (5 mg/ml) was added into each well. After incubation at 37°C for 4 h, the medium was removed, 100 μl of DMSO was added to each well and absorbance was read at 490 nm using a microplate reader (BMG Labtech GmbH, Ortenberg, Germany). The experiments were performed three times, and the mean absorbance values were calculated. The results are expressed as the percentage of inhibition that produced a reduction in the absorbance by PE treatment compared with the control group (not treated with PE).

### Cell cycle analysis

In total, 1×10^6^ cells were synchronised by exposure to medium with a low concentration of FBS for 24 h to induce cell cycle arrest. The culture medium was then replaced with nutrient-rich medium, and the cells were treated with various concentrations of PE for 24 h. The cells were collected by trypsinisation and washed twice with cold PBS. The cells were then fixed with 75% cold ethanol at 4°C for 24 h. Prior to analysis, the cells (1×10^5^) were labelled with propidium iodide (PI) (1 mg/ml) in the presence of 1% RNase A for 30 min. The cells were sorted in a FACS Calibur flow cytometer using BD Cell Quest software (San Jose, CA, USA).

### Apoptotic assay

Cell apoptosis was determined using an Annexin V-FITC Apoptosis Detection kit I according to the manufacturer’s protocol. SMMC-7721 cells were cultured at 1×10^6^ cells/ml in 6-well plates for 24 h. The culture medium was replaced with fresh medium, and the cells were treated with indicated concentrations of PE for 24 h. After digesting with 0.25% trypsin-EDTA for 3 min, the cells were collected and centrifuged at 71.55 × g for 8 min. The pellets were then washed twice with cold PBS. Approximately 1×10^5^–1×10^6^ cells were resuspended in 100 μl 1X binding buffer and were transferred to a sterile flow cytometry glass tube. The cells were incubated with 5 μl Annexin V-FITC and 5 μl of 20 μg/ml PI in the dark at room temperature for 15 min. The apoptotic cells were analysed using a flow cytometer (FACS Calibur; BD Biosciences). Annexin V-FITC and PI emissions were detected in the FL 1 and FL 2 channels.

### Measurement of ΔΨm

ΔΨm was analysed using a rhodamine 123 assay as previously described (24). Rhodamine 123 can be selectively absorbed by mitochondria and is proportional to the ΔΨm (25). Briefly, the cells were seeded in 24-well plates at 2×10^5^ cells/well. Subsequent to PE treatment for 24 h, the cells were washed twice with cold PBS and incubated with cold PBS containing 1 μl of 10 μg/μl rhodamine 123 at 37°C for 30 min. The fluorescent intensity of rhodamine 123 in the mitochondria was detected using a fluorescence microplate reader at an excitation wavelength of 505 nm and an emission wavelength of 527 nm. The data were expressed as a percentage of the control.

### Confocal microscopy

For immunofluorescence studies, SMMC-7721 cells were seeded in 6-well plates for 24 h. The cells were washed twice with ice-cold PBS and fixed with 75% cold ethanol for 30 min at 4°C. After washing with PBS three times, the cells were incubated with 0.5% Triton X-100 for 25 min. The cells were blocked by incubation with 10% goat serum in PBS containing 0.3% Triton X-100 and 0.5% BSA at room temperature for 20 min, followed by incubation with a mouse monoclonal antibody against Bcl-2, Bax, caspase-3, phospho-Erk1/2, phospho-Stat1 and phospho-Stat2 (1:100 in PBS) at 4°C overnight. Cells were then incubated with FITC-conjugated goat anti-mouse IgG (1:100 in PBS) for 2 h at 37°C in a humidified atmosphere. After washing three times with PBS, the fluorescent images of cells were collected using a Leica TCS SP2 laser scanning confocal microscope (Leica).

### Western blotting

SMMC-7721 cells (1×10^6^) were incubated in a 6-well plate for 24 h and treated with different concentrations of PE for the indicated time periods. Whole-cell lysates were prepared from PE-treated and untreated cells in RIPA buffer (20 mM Tris-HCl pH 7.5, 120 mM NaCl, 1.0% Triton X-100, 0.1% SDS, 1% sodium deoxycholate, 10% glycerol, 1 mM EDTA and 1% protease inhibitor cocktail). Following centrifugation at 12,879.36 × g at 4°C for 5 min, the protein concentrations in the supernatants were determined after the cells were centrifuged at 12,879.36 × g at 4°C for 5 min. Equal amounts of protein (50 μg) in whole-cell lysates were mixed with reducing sample buffer (0.92 M Tris-HCl pH 8.8, 1.5% SDS, 4% glycerol, and 280 mM 2-ME), and the samples were boiled at 99°C for 8 min, separated on SDS-polyacrylamide gels and transferred to polyvinylidene difluoride (PVDF) membranes. The membranes were blocked in blocking buffer (Tris-buffered saline containing 3% BSA, 20 mM NaF, 2 mM EDTA, and 0.2% Tween-20) for 2 h at 37°C. The membrane was then incubated for 2 h with the appropriate primary antibody at 37°C. The primary antibodies were used at the following dilutions: 1:800 for phospho-Erk1/2, phospho-Stat1 or phospho-Stat2, 1:500 for Bcl-2 and 1:3,000 for GAPDH. The membrane was washed three times with TBST buffer (Tris-buffered saline containing 20 mM NaF, 2 mM EDTA, and 0.2% Tween-20), incubated for 60 min with HRP-conjugated secondary antibodies and washed three times with TBST buffer. Detection was achieved by chemical fluorescence following an enhanced chemiluminescence (ECL) western blotting protocol (Amersham Biosciences, Piscataway, NJ, USA). The image capture and analysis were performed using a GeneGnome imaging system (Syngene, Frederick, MD, USA).

### Statistical analysis

Data are presented as the means ± SD of at least three independent experiments that were obtained from different cultures. All the analyses were performed using the SPSS software version 15.0. An analysis of variance (ANOVA) was used to test for significant differences between the groups, which was followed by the Dunnett’s test for multiple comparisons. Differences were considered significant when the calculated P-values were <0.05.

## Results

### PE treatment decreases viability in SMMC-7721 cells

**Figure 1:**
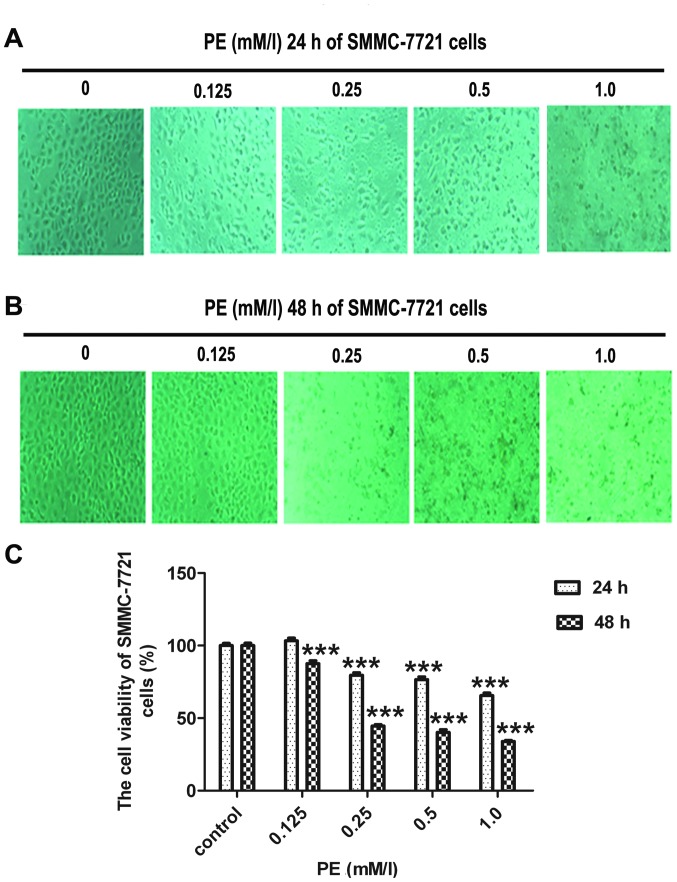
The effect of phosphatidylethanolamine (PE) on SMMC-7721 cells viability. (A) Representative light microscopy image of SMMC-7721 cells. SMMC-7721 cell culture morphology was assessed by light microscopy after incubation for 24 h in the presence of PE. (B) Representative light microscopy image of SMMC-7721 cells. SMMC-7721 cell culture morphology was assessed by light microscopy after incubation for 48 h in the presence of PE. (C) Viability of PE on SMMC-7721 cells. Cells were treated with different concentrations of PE (0, 0.125, 0.25, 0. 5 and 1.0 mM/l) for 24 and 48 h, and the cell viability was determined by MTT assay. The results were expressed as the percentage of cell growth relative to the control group. The data were represented as the mean ± SD (n=3). ^***^P<0.001 vs. the control group.

The effects of PE on the viability of SMMC-7721 cells were assessed using the MTT uptake method. PE inhibited the growth of SMMC-7721 cells in a concentration- and time-dependent manner (Fig. 1). In SMMC-7721 cells, the viability value was 80% after treatment with 0.25 mM/l PE for 24 h, whereas for treatment with 0.5 and 1.0 mM/l PE after 24 h, the values were 77 and 66%, respectively. After 48 h, in response to treatment with 0.125, 0.25, 0.5 and 1.0 mM/l PE, the viability values of SMMC-7721 cells decreased to 88, 45, 40 and 34%, respectively.

Direct observation using an inverted microscope revealed numerous morphological changes in the cells that were treated with PE (Fig. 1B and C). The untreated cells exhibited a regular and healthy shape. After incubation with PE, the cellular morphology was severely distorted and polyhedral or irregular and even grew in a tightly connected manner.

### PE induced a G0/G1 cell cycle arrest

**Figure 2:**
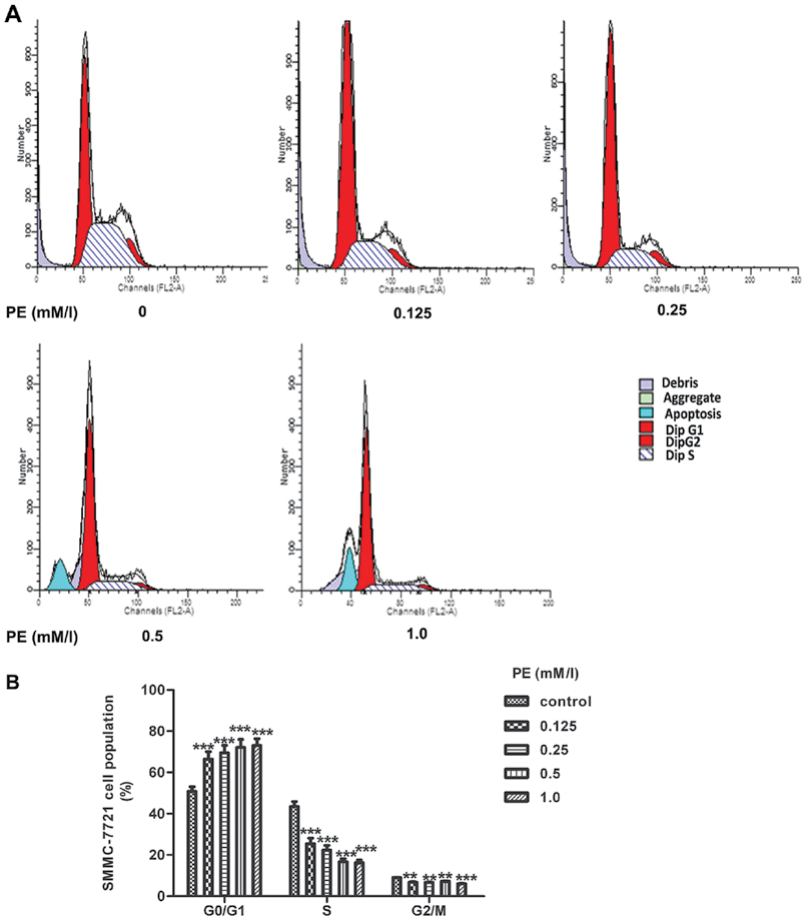
Effects of phosphatidylethanolamine (PE) on the SMMC-7721 cell cycle. (A) Representative images of FACS analysis. SMMC-7721 cells were treated with different concentrations of PE for 24 h. Then, the cell cycle distribution was analyzed using flow cytometry. (B) Statistical analysis of the percentages of cells in the various phases. Tests were performed independently in triplicate (n=3). Data are presented as the means ± SD. ^**^P<0.01 and ^***^P<0.001 vs. the control group.

To assess whether the PE-induced cell growth inhibition was due to arrest at a specific point of the cell cycle, SMMC-7721 cells were synchronised by exposure to medium with a low FBS concentration for 24 h until starvation. The cells were then returned to the culture nutrient-rich medium, which stimulated cell proliferation, with different concentrations of PE for 24 h. The cells were collected for flow cytometric analysis. As shown in Fig. 2B, the PE-treated cells exhibited a dose-dependent accumulation of G0/G1 phase-arrested cells compared with the corresponding untreated cells. At 1.0 mM/l, PE significantly increased cell cycle arrest during the G0/G1 phase of SMMC-7721 cells to 77±0.40%, whereas the percentage of SMMC-7721 cells in the G0/G1 phase in the control group was only 53±0.28%. This increase in the G1 cell population was mostly at the expense of the S and G2/M phase cell populations.

### PE-induced apoptosis

**Figure 3:**
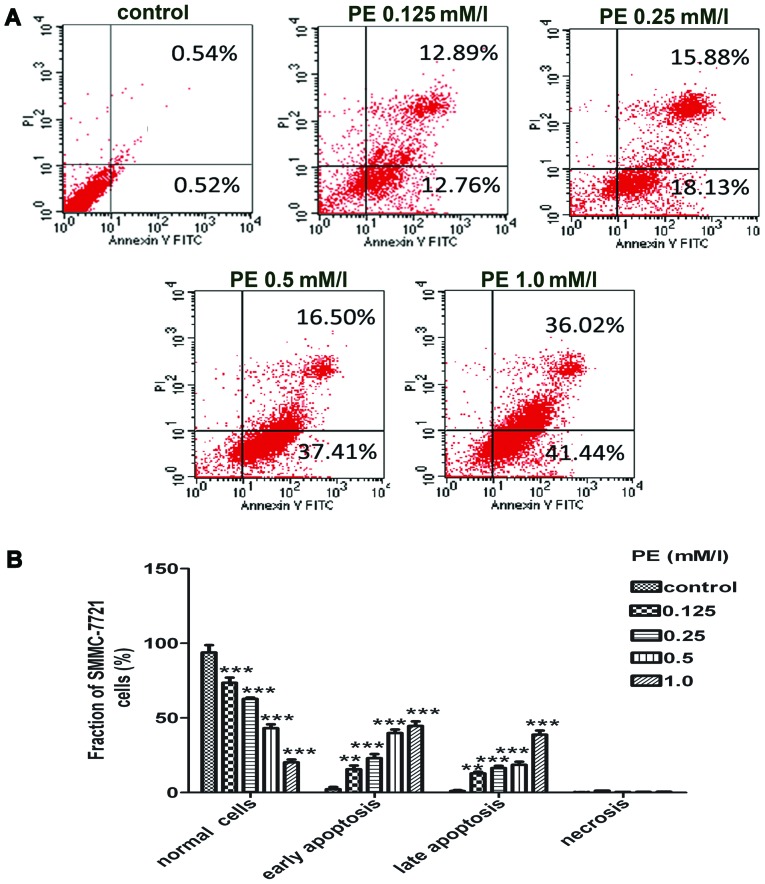
Detection of phosphatidylethanolamine (PE)-induced apoptosis in SMMC-7721 cells. (A) The representative images of flow cytometry analysis were shown. After the cells were treated with PE (0, 0.125, 0.25, 0.5 and 1.0 mM/l) for 24 h, the cells were stained with FITC-conjugated Annexin V and propidium iodide (PI), followed by flow cytometric analysis. Cell populations with Annexin V^−^/PI^−^, Annexin V^+^/PI^−^, Annexin V^+^/PI^+^, Annexin V^−^/PI^+^ were regarded as living, early apoptotic, late apoptotic and necrotic cells, respectively. (B) Statistical analysis of the percentages of the apoptotic cells. The results were presented as the mean ± SD of three independent experiments performed. ^**^P<0.01 and ^***^P<0.001 vs. the control group.

To examine the degree of apoptosis that was induced by PE, we used Annexin V-FITC and PI to distinguish apoptotic cells from necrotic cells. Annexin V is a binding protein with a strong affinity and selectivity for phosphatidylserine, which appears on the cell surface as a general indicator of apoptosis. However, the translocation of phosphatidylserine to the cell surface also occurs during necrosis (26). Therefore, measuring Annexin V binding to the cell surface was performed in conjunction with PI staining. Early apoptotic cells were identified by positive Annexin V-FITC and negative PI staining, whereas cells that were in late apoptosis or necrotic cells were positive for both Annexin V-FITC and PI. Viable cells were negative for both Annexin V-FITC and PI. The results were interpreted as follows: cells in the lower left quadrant (Annexin V^−^/PI^−^) were considered to be viable cells, cells in the lower right quadrant (Annexin V^+^/PI^−^) were considered early apoptotic cells, cells in the upper right quadrant (Annexin V^+^/PI^+^) were considered late apoptotic cells, and cells in the upper left quadrant (Annexin V^−^/PI^+^) were considered necrotic cells. The total apoptotic rate was calculated as the rate of cells in the lower right quadrant (Annexin V^+^/PI^−^) plus the rate of cells in the upper right quadrant (Annexin V^+^/PI^+^). As shown in Fig. 3, PE increased the proportion of SMMC-7721 cells that were stained with both Annexin V and PI in a concentration- and time-dependent manner. The total percentage of apoptotic and necrotic SMMC-7721 cells in the untreated cells increased from 3±0.21 to 25±3.93, 35±3.81, 55±3.46, and 77±1.70%, when the cells were treated with 0.125, 0.25, 0.5 and 1.0 mM/l PE, respectively, for 24 h. The early apoptotic rate was 0.76±0.06% in the control group, whereas this rate markedly increased to 15±0.76, 21±1.08, 37±0.84, and 41±0.52% in the experiment groups of treated with 0.125, 0.25, 0.5 and 1.0 mM/l PE, respectively for 24 h. These results showed that PE efficiently induced apoptosis in SMMC-7721 cells.

### Evaluation of ΔΨm damage

**Figure 4:**
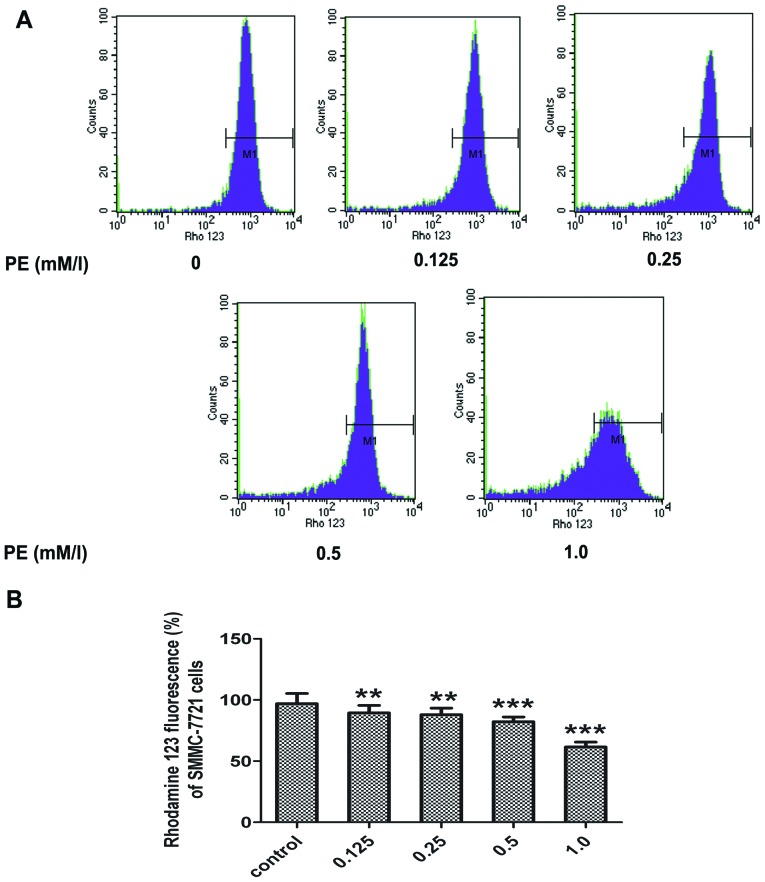
Participation of mitochondria in apoptosis induction. (A) Representative images of flow cytometric analysis showing changes in the mitochondrial membrane potential (ΔΨm). SMMC-7721 cells were treated with 0, 0.125, 0.25, 0.5 and 1.0 mM/l phosphatidylethanolamine (PE) for 24 h, and the ΔΨm was measured by FCM using rhodamine 123 dye. The control cells show a high membrane potential, whereas the experiment cells show a low membrane potential. (B) Statistical analysis of ΔΨm examined by using rhodamine 123 staining and flow cytometric analysis. Data were obtained from three independent experiments. ^**^P<0.01 and ^***^P<0.001 vs. the control group.

Mitochondrial dysfunction is an important characteristic of apoptotic cell death. ΔΨm perturbation under PE treatment was examined. To evaluate the changes in the ΔΨm, a mitochondria-specific dye rhodamine 123 was used. As shown in Fig. 4, following the PE treatment of SMMC-7721 cells for 24 h, the level of the ΔΨm decreased compared with the control group. A dose-dependent reduction in ΔΨm was also observed in the PE-treated cells. These data indicated that PE-induced apoptosis was accompanied by a collapse in the ΔΨm.

### Detection of caspase-3 activity

Since caspase activation is a major step in apoptosis, we studied the involvement of caspase activation in PE-stimulated apoptosis in SMMC-7721 cells using immunofluorescence analysis. As shown in Fig. 6, PE treatment caused the levels of caspase-3 to increase in a concentration-dependent manner. Exposure to 0.125, 0.25, 0.5 and 1.0 mM/l PE for 24 h resulted in 1.2-, 1.6-, 2.0- and 2.4-fold increases in caspase-3 activity.

### Evaluation of Bax and Bcl-2

**Figure 5:**
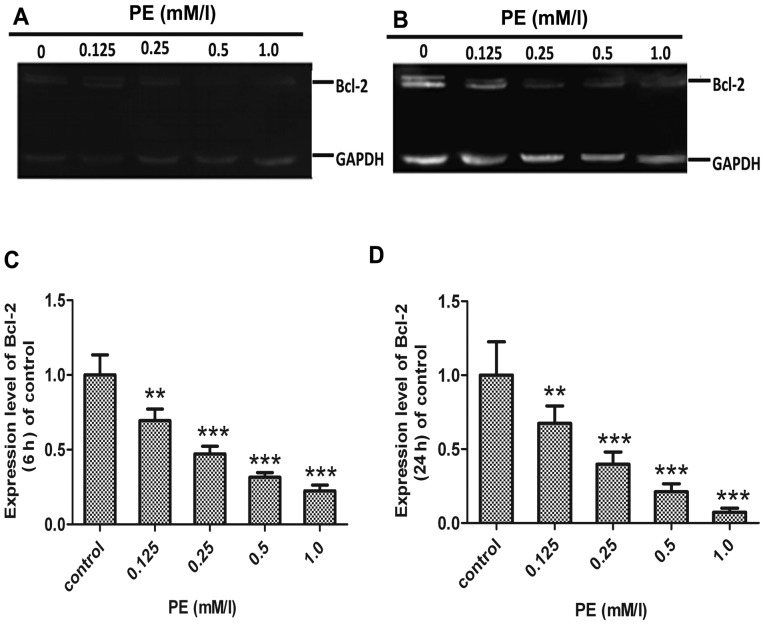
Effects of phosphatidylethanolamine (PE) treatment on Bcl-2 expression in SMMC-7721 cells. The cells were seeded in a 6-well plate and then exposed to PE for the indicated times of 6 and 24 h. After preparing whole-cell lysates or cytosolic extracts, the levels of Bcl-2 and GAPDH were detected by immunoblotting. (A) The expression of Bcl-2 was detected at 6 h with GAPDH as a loading control. (B) The expression of Bcl-2 was detected at 24 h with GAPDH as a loading control. (C and D) Statistical analysis of (A and B), respectively. The relative density of Bcl-2/GAPDH was compared to the control group. Data are presented as the means ± SD (n=3). ^**^P<0.01 and ^***^P<0.001 vs. the control group.

**Figure 6:**
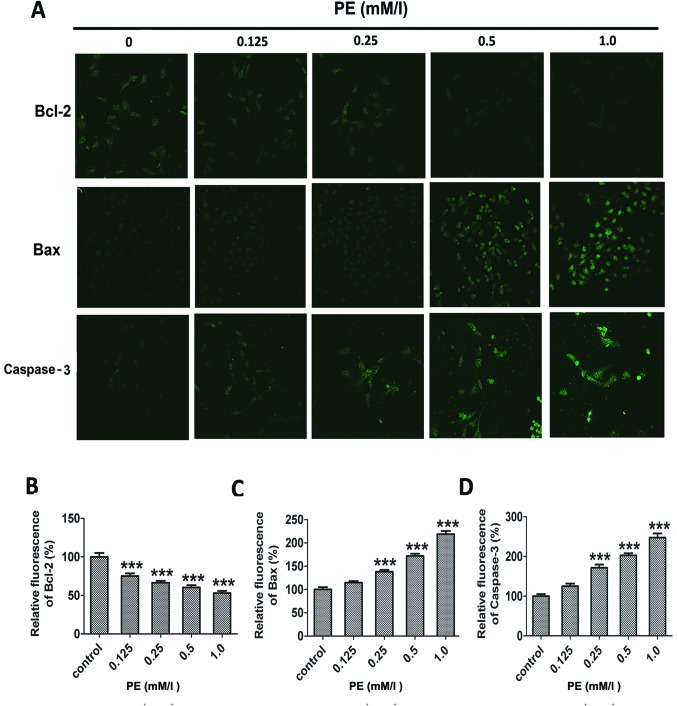
Effects of phosphatidylethanolamine (PE) on the expression of apoptosis-related proteins. (A) Representative images of immunofluorescence analysis of the expression of anti-apoptotic Bcl-2, pro-apoptotic Bax and Caspase-3 in SMMC-7721 cells that were incubated with PE for 24 h. (B–D) Statistical analysis of the expression of Bcl-2, Bax and Caspase-3, respectively. The relative fluorescence intensity was compared to the control group. All the tests were performed independently in triplicate (n=3). Data are presented as the means ± SD. ^***^P<0.001 vs. the control group.

The pro-apoptotic protein, Bax, has been reported to translocate from cytosol to mitochondria following the exposure of cells to apoptotic stresses. The anti-apoptotic protein Bcl-2 is known to prevent Bax redistribution to the mitochondria, caspase activation and apoptosis (27). In the present study, to detect whether regulation of Bax and Bcl-2 was involved in PE-induced apoptosis, we examined the changes in Bax and Bcl-2 using immunofluorescence and western blotting in SMMC-7721 cells. As shown in Figs. 5 and 6, Bax expression was upregulated, whereas expression of Bcl-2 was downregulated in SMMC-7721 cells after PE exposure. Therefore, PE treatment induced apoptosis, which was accompanied by the dose-dependent downregulation of Bcl-2 and upregulation of Bax.

### Effects of PE on MAPK phosphorylation and Stat signalling

**Figure 7:**
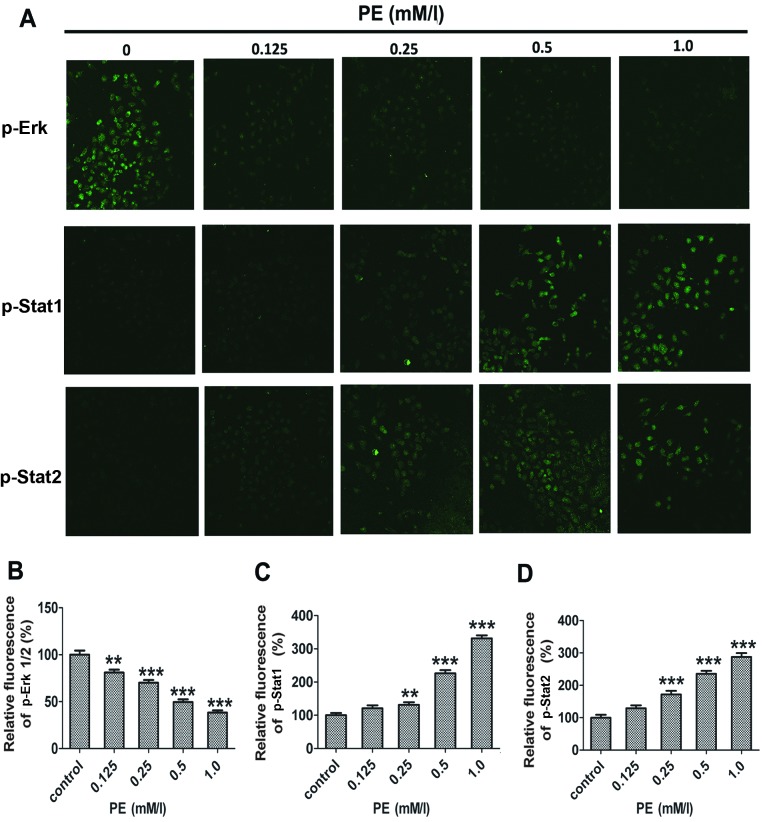
Effects of phosphatidylethanolamine (PE) on the phosphorylation of Erk1/2 and signal transducers and activators of transcription factors (Stats) signalling. (A) Representative images of immunofluorescence analysis of the expression of P-Erk1/2, p-Stat1 and p-Stat2 in SMMC-7721 cells that were incubated with PE for 24 h. (B–D) Statistical analysis of the expression of P-Erk1/2, p-Stat1 and p-Stat2, respectively. The relative fluorescence intensity was compared to the control group. The tests were performed independently in triplicate (n=3). Data are presented as the means ± SD. ^**^P<0.01 and ^***^P<0.001 vs. the control group.

**Figure 8:**
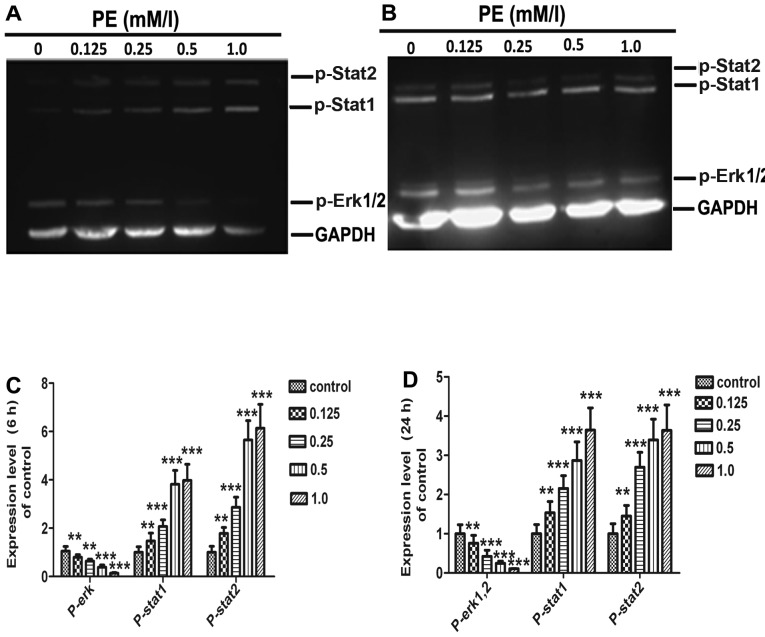
Effects of phosphatidylethanolamine (PE) on the phosphorylation of Erk1/2 and signal transducers and activators of transcription factor (Stats) signalling. The cells were plated in 6-well plates and exposed to PE for 6 and 24 h. After preparing the whole-cell lysates, the phosphorylated protein levels of Erk1/2, Stat1, Stat2 and GAPDH were determined by immunoblotting. (A) The expression of p-Erk1/2, p-Stat1 and p-Stat2 was detected at 6 h with GAPDH as a loading control. (B) The expression of p-Erk1/2, p-Stat1 and p-Stat2 was detected at 24 h with GAPDH as a loading control. (C and D) Statistical analysis of (A and B), respectively. The relative density of p-Erk1/2, p-Stat1 and p-Stat2/GAPDH was compared to the control group. The tests were performed independently in triplicate (n=3). Data were presented as the means ± SD. ^**^P<0.01 and ^***^P<0.001 vs. the control group.

MAPKs are known to play an important cellular regulatory role (12,14). MAPKs and signal transducers and activators of transcription factor (Stats) signalling pathways regulate cell proliferation, survival, and differentiation (28). To investigate the involvement of MAPKs and Stats in PE-induced apoptosis in SMMC-7721 cells, we examined changes in the phospho-Erk and phospho-Stat activities of PE-treated cells using western blotting. As shown in Figs. 7 and 8, Erk phosphorylation decreased continuously in a time- and dose-dependent manner; however, the level of Stat1/2 phosphorylation increased.

## Discussion

The results of the MTT assay employed in the present study showed that PE treatment suppressed the viability of the SMMC-7721 human hepatic cancer line. The effect was gradually enhanced with increasing PE concentrations (0.125, 0.25, 0.5 and 1.0 mM/l). Moreover, we observed a marked inhibitory effect when SMMC-7721 cells were treated with PE for 48 h compared with 24 h. This result suggests that PE inhibited the viability of SMMC-7721 cells in a dose- and time-dependent manner. To determine the mechanisms that lead to the loss of SMMC-7721 cell proliferation by PE, the effects of PE treatment on cell cycle arrest were examined. An analysis of the cell cycle following the treatment of SMMC-7721 cells with different concentrations of PE showed a higher number of cells in the G0/G1 phase in a dose-dependent manner compared to the untreated cells. These results suggest that PE inhibited cell proliferation via a G0/G1 phase arrest. We also found that PE inhibited cell proliferation via a G0/G1 phase arrest in HEK-293 cells (data not shown). To examine whether apoptosis is involved in PE-induced cell death in SMMC-7721 and HEK-293 cells, we investigated the apoptotic effect of PE using flow cytometry for Annexin V/PI staining. We found that PE treatment directly induced apoptotic death in SMMC-7721 and HEK-293 cells. Accordingly, we considered that exogenous PE-induced apoptosis was responsible for its cytotoxicity in SMMC-7721 and HEK-293 cells. Although PE externalisation has been identified as a molecular marker of apoptosis, little is known regarding the effects of exogenous PE on apoptosis. We hypothesized that PE is stimulated by PE externalisation of the cytomembrane and that PE externalisation may be indicative of apoptosis and induce cell apoptosis *in vitro*.

Mitochondria are the critical mediators of apoptosis in the intrinsic pathway (29). The changes in the ΔΨm and mitochondrial dysfunction are considered an early event in apoptosis (30,31). On receiving a death signal, the mitochondrial membrane is disrupted, and cytochrome *c* is released from mitochondria into the cytosol (30). Bcl-2 family proteins have been shown to play an important role in the regulation of mitochondria-mediated apoptosis, and are the upstream regulators of the ΔΨm (32). The pro-apoptotic member Bax translocates to the mitochondrion and integrates into the OMM, where Bax promotes the excretion of cytochrome *c* into the cytosol and the disruption of ΔΨm, whereas anti-apoptotic protein Bcl-2 prevents this process by preserving mitochondrial integrity (33). Bcl-2 protects cells against apoptosis and modulates OMM permeability and the release of cytochrome *c* (34–36). It has been suggested that Bax induces a decrease in the membrane potential of mitochondria, leading to an increase of mitochondrial membrane permeability and the release of cytochrome *c* from mitochondria (37–39). Thus, the balance between Bax and Bcl-2 is crucial in sustaining apoptosis in the intrinsic pathway (40). Cytochrome *c*, which is released from mitochondria leads to the subsequent activation of downstream caspases, such as caspase-3 (41). Caspase-3, which is a downstream effector in the caspase cascade, is considered an essential executor for mitochondrial-dependent apoptotic pathways (42,43). In this study, PE treatment decreased the ΔΨm in SMMC-7721 and HEK-293 cells (data not shown). To verify PE-induced apoptosis, we determined the expression of Bax, Bcl-2 and caspase-3 in SMMC-7721 cells by western blotting. We found that PE increased caspase-3 expression in a dose-dependent manner in SMMC-7721 cells. This result suggests that the mitochondrial pathway is potentially involved in PE-induced cell apoptosis. Our results also show that PE treatment upregulated the expression of Bax and downregulated the level of Bcl-2 in a dose-dependent manner, which eventually leads to an increase in the ratio of Bax/Bcl-2 protein levels. We demonstrated that Bax/Bcl-2 signalling pathways may be involved in PE-induced apoptosis, which has been accompanied by conspicuous reduction in the ΔΨm.

MAPKs are key mediators that transduce extracellular signals from the membrane to the nucleus (44). As a member of the MAPK family, Erk is important in the regulation of cell growth and mediates a survival response that counteracts cell death (12). However, other studies have reported that the activation of Erk is associated with apoptosis (45,46). Therefore, the role of MAPK signalling depends on the stimuli and cell type (47). In the present study, whether Erk activation was involved in the PE-induced apoptotic cell death was evaluated in SMMC-7721 cells that were treated with PE. The results showed that PE treatment markedly suppressed Erk activation, which indicated the involvement of Erk pathways in PE-induced apoptotic death in SMMC-7721 cells. Tamura *et al* reported that Erk, as the responsible kinase for the phosphorylation of Bcl-2, exerts an effect on the anti-apoptotic function of Bcl-2 in human tumour cell lines (48). Accordingly, exogenous PE-induced apoptosis may be related to the downregulation of Erk and to the repression of Bcl-2. Erk should be an upstream regulator of Bcl-2. Results of previous studies have shown that the activation of JNK and P38 is necessary for cancer cell death which is initiated by a variety of anti-cancer agents and that, notably, the JNK pathway plays an important role in the activation of the mitochondrial-dependent apoptotic pathway (49,50). Whether JNK activation is involved in the mitochondrial apoptotic pathway by PE treatment in SMMC-7721 cells may require further elucidation.

The Stats are a family of latent cytoplasmic transcription factors that mediate intracellular signalling that is initiated at cytokine cell-surface receptors and is transmitted to the nucleus (51). After the ligation of cytokine receptors, Stats become phosphorylated by receptor kinases, dimerise and translocate to the nucleus, where these molecules modulate the expression of Stat-responsive genes (19). By regulating the target gene expression, Stat proteins have been shown to play a major role in mediating extensive biological processes, such as cell proliferation, survival, apoptosis and differentiation (37–39). Stat1 is partially phosphorylated by the Erk pathway (16), and phosphorylation of Stat1 appears to be required for maximal transcriptional activity (18). It has been shown that Stat1 may induce apoptotic or cell cycle checkpoint responses following various stressful stimuli (16,19–22). A previous study also reported that Stat1 functionally promoted apoptosis and tumour suppression (52). In this study, we observed that PE treatment significantly increased the activation of Stat1/Stat2 in SMMC-7721 cells (Figs. 7 and 8), suggesting that the Stat1/Stat2 pathway may be involved in PE-induced SMMC-7721 cell apoptosis. It has been shown that constitutively high activities of Erk and PI3K-AKT in LU1205 cells inhibit Stat-transcriptional activities via their effects on JAK2 (53). Another study showed that in U3A-ST1 cells that constitutively express Stat1, IFNγ, which is a known activator of Stat1, reduces the basal expression of the Bcl-2 promoter (23). Therefore, PE may exert its inhibitory effects on Bcl-2 through the inhibition of the Erk pathway and through the activation of the Stat1/2 pathway, which would eventually promote SMMC-7721 cell apoptosis. A recent study proved that testicular lumicrine factors protect the cells of the initial segment by activating the Erk pathway, repressing Stat pathways, and preventing apoptosis (54). This result may support our conclusions from another viewpoint.

In conclusion, our results have demonstrated the potential apoptotic effect of exogenous PE on SMMC-7721 cells. We hypothesized that PE may induce a decrease in the membrane potential of the mitochondria via the upregulation of the ratio of Bax/Bcl-2 protein levels, which subsequently leads to caspase-3-dependent apoptosis. In addition, the inhibition of Erk and the activation of Stat1/2 signalling may be involved in the PE-induced apoptosis of SMMC-7721 cells.
